# Acceleration of biodetoxification on dilute acid pretreated lignocellulose feedstock by aeration and the consequent ethanol fermentation evaluation

**DOI:** 10.1186/s13068-016-0438-9

**Published:** 2016-01-26

**Authors:** Yanqing He, Jian Zhang, Jie Bao

**Affiliations:** State Key Laboratory of Bioreactor Engineering, East China University of Science and Technology, 130 Meilong Road, 200237 Shanghai, China

**Keywords:** Biodetoxification, Inhibitors, *Amorphotheca resinae* ZN1, Aeration, Simultaneous saccharification and ethanol fermentation (SSF), Lignocellulose

## Abstract

**Background:**

Biodetoxification by the fungus *Amorphotheca resinae* ZN1 provides an effective way of inhibitor removal from pretreated lignocellulose feedstock and has been applied in the process of ethanol, biolipids, and lactic acid production. However, the long-time used and the consumption of considerable xylose in the pretreated materials reduced the process efficiency. The improvements of biodetoxification should be made to enhance the production of biochemical from lignocellulosic materials.

**Results:**

This study reported an acceleration method of *A. resinae* ZN1-based biodetoxification on the corn stover (CS) feedstock pretreated using dry dilute acid pretreatment. Under proper aeration and well-mixing condition, the conversion rate of furfural, 5-hydroxymethylfurfural (HMF), acetic acid, and typical phenolic compounds were significantly accelerated by more than twofolds faster, which resulted in the reduction of biodetoxification time from 96 h in the conventional process to 36 h. Simultaneous saccharification and ethanol fermentation assay on accelerated biodetoxification of the dry dilute acid pretreated CS feedstock achieved the similar ethanol titer (48.56 g/L of 36 h’ accelerated biodetoxification vs. 50.00 g/L of 4 days’ conventional biodetoxification) and yield (58.10 vs. 59.63 %). Substrate priority of inhibitors to sugars by *A. resinae* ZN1 was discovered and considerable xylose was reserved in the accelerated biodetoxification. Cell growth of *A. resinae* fungus in liquid medium and on pretreated CS solids revealed that the enhanced aeration enhanced the biodetoxification rate rather than the cell growth rate. Accelerated inhibitor conversion might come from the increased supply of cofactors of nicotinamide adenine dinucleotide or nicotinamide adenine dinucleotide phosphate from the step of aldehyde inhibitors to the corresponding acids, instead of cell mass increase.

**Conclusion:**

Accelerated biodetoxification reduced the period of biodetoxification and retained the xylose components in the pretreated CS, which provided a practical method on improving process efficiency for cellulosic ethanol production from severe pretreated lignocellulose feedstock.

**Electronic supplementary material:**

The online version of this article (doi:10.1186/s13068-016-0438-9) contains supplementary material, which is available to authorized users.

## Background

Pretreatment is the key step to overcome the biorecalcitrance of lignocellulose to obtain fermentable sugar formation in enzymatic hydrolysis step. During pretreatment processing, over-degradation of partial cellulose, hemicellulose, and lignin leads to the generation of various furan derivatives, organic acids, and phenolic compounds. These compounds severely inhibit consequent fermenting microbes [1]. Therefore, the removal of inhibitory compounds or “detoxification” is a prerequisite step for well growth and metabolism of fermenting strains to produce target biofuels and biochemicals. Available detoxification methods include water washing [2], overliming using Ca(OH)_2_ [3], ion-exchange, activated charcoal absorption [4], but these physical or chemical methods lead to the generation of huge waste water and loss of soluble sugars [5].

In recent few years, a new detoxification method using specific inhibitor degrading microorganisms demonstrated advantages of mild condition, low cost, high degradation efficiency, low energy consumption [6–9]. Various microorganisms have been isolated and applied to biodetoxification of inhibitors from pretreatment, including bacteria [6, 10–15], yeasts [16–18], and fungi [7, 19–24]. Among these studies, the fungus strain *Amorphotheca resinae* ZN1 performed biodetoxification on solid pretreated lignocellulose feedstock then applied to the consequent enzymatic hydrolysis and fermentation [7] for the production of ethanol [25], lipids [26], and lactic acid [27] with high product yield and zero waste water generation obtained. However, approximately 4–7 days of biodetoxification time and loss of xylose released from pretreatment are the two major technical barriers on reducing the overall process efficiency [7, 25].

In our previous study, the degradation of furfural and 5-hydroxymethylfurfural (HMF) in the synthetic medium by *A. resinae* ZN1 was improved when the constant aeration was kept during the culture. Thus this method was applied in the biodetoxification of pretreated corn stover (CS) to overcome the two drawbacks of biodetoxification method. Interestingly, the acceleration of biodetoxification of freshly pretreated CS material was realized by proper aeration and well-mixing approaches. Biodetoxification time was reduced from 96 h in the conventional biodetoxification to 36 h in the accelerated biodetoxification under the aeration rate of 1.00 vvm. Meanwhile, most of xylose released from dilute acid pretreatment was maintained during the shortened biodetoxification time because of the priority of inhibitor compounds to xylose by *A. resinae* ZN1. The present accelerated biodetoxification method provided an important solution for industrial application of biodetoxification approach in biorefining of lignocellulose for biofuels and biochemical production.

## Results and discussion

### Biodegradation of inhibitors on solid pretreated CS by *A. resinae* ZN1

**Fig. 1:**
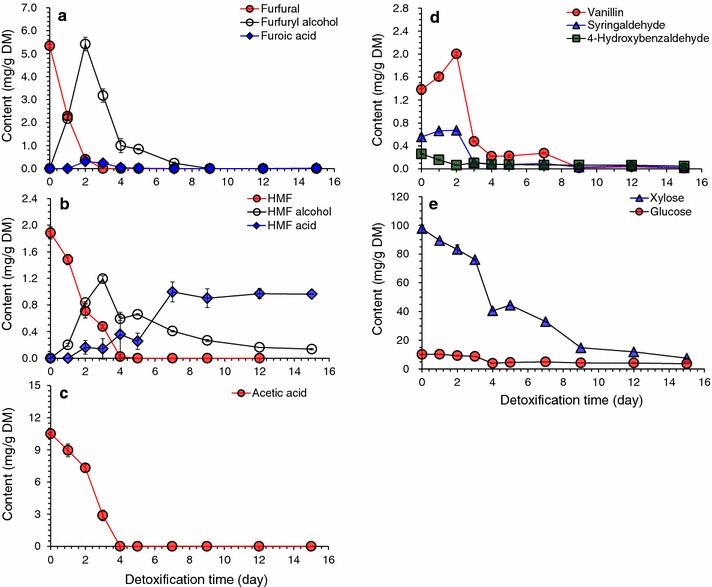
Biodetoxification of dry dilute acid pretreated corn stover by *A. resinae* in conventional solid state mode. The biodetoxification was carried out in a plastic box with 2 kg pretreated corn stover loading at 28 °C, pH 5.5, and 10 % (*w/w*) inoculum of solid seed. **a** Furfural conversion; **b** HMF conversion; **c** Acetic acid conversion; **d** Phenolics conversion; **e** Sugars consumptions

Inhibitor degradation by *A. resinae* ZN1 has been investigated in synthetic medium [28, 29]. The detailed degradation in real pretreated lignocellulose materials has not yet completely profiled although the biodetoxification method had been applied in the pretreated materials such as CS, wheat straw, rape straw, and rice straw as described in [7]. In this study, the inhibitor conversion performance in the pretreated CS was examined. After the seed mycelia of *A. resinae* ZN1 was inoculated onto the pretreated CS, the major representative inhibitors in the pretreated CS were monitored during biodetoxification process (Fig. 1), including two furan aldehydes, furfural, and HMF; one weak acid, acetic acid; and three phenolic compounds, 4-hydroxybenzaldehyde representing H group of lignin compounds, syringaldehyde representing S group, and vanillin representing G group.

Furfural was quickly converted to the corresponding furfuryl alcohol, then to the potential intermediate furoic acid at low concentration and quickly consumed (Fig. 1a). HMF was converted to HMF alcohol (2,5-furandimethanol) and HMF acid (5-Hydroxymethyl-2-furoic acid) (Fig. 1b). The tendency of furfural and HMF was similar to that in the synthetic medium [28, 29]. The sum of furfural or HMF with their corresponding metabolites during biodetoxification was approximately in mass balance to the starting furfural or HMF. Approximately, the complete degradation of furfural took 3 days and HMF took 4 days for the pretreated CS material. Acetic acid completely degraded within 4 days with no secondary metabolites detected (Fig. 1c). The conversion rate of acetic acid was accelerated when furfural was reduced to low content at 2 days. The conversion of the three phenolic aldehydes shows that vanillin and syringaldehyde increased in the first 3 days and then quickly degraded to a low level, while the relatively low titer of 4-hydroxybenzaldehyde kept decreasing (Fig. 1d). The degradation rate of vanillin and syringaldehyde accelerated after the complete conversion of furfural at 3 days. The result suggests that the decrease of furfural to a certain point triggered the quick degradation of other inhibitors such as HMF, acetic acid, phenolic aldehydes. On the other hand, xylose consumption in the pretreated CS rate accelerated when furfural was completely removed: only 4.7 mg/g DM per day for xylose in the first 3 days, but reached 34 mg/g DM per day for xylose at 4th day and quickly consumed out in the next few days, indicating that furfural decrease also triggered the quick consumption of glucose and xylose. Based on the results, the complete removal time of furfural by *A. resinae* ZN1 was defined as the ending point of biodetoxification for the present pretreated CS material.

After the conventional biodetoxification, the CS feedstock was sent to simultaneous saccharification and ethanol fermentation (SSF) at 30 % (*w/w*) solids content and 15 FPU/g DM cellulase dosage. Corn stover hydrolysate without biodetoxification treatment contained 0.75 g/L of furfural, 0.53 g/L of HMF, 0.57 g/L of vanillin, and 0.26 g/L of syringaldehyde, and the high inhibitor content resulted in a low glucose generation in the 12 h’ pre-hydrolysis (41.99 g/L of glucose) and ethanol titer in the SSF (5.31 g/L) (Table 1). After 1 day’s detoxification, furfural was decreased to half of the original concentration (0.35 g/L), the glucose concentration in the pre-hydrolysis (71.57 g/L of glucose) and the ethanol titer in the SSF (46.16 g/L) were significantly increased. As the biodetoxification time on pretreated CS feedstock prolonged, the glucose concentration in the pre-hydrolysis and the ethanol titer in the SSF increased correspondingly. Although the main inhibitors such as furans, acetic acid, and three phenolic compounds were degraded, the kinds of residual phenolic compounds were various and most of them were hard to separate from CS hydrolysate for detection. These compounds might be a potential inhibitor of cellulase. The prolonging of biodetoxification period decreased the level of these compounds leading to the increase of glucose concentration after pre-hydrolysis and ethanol titer. Four days’ detoxification time led to the better fermentation performance on ethanol titer (50.00 g/L) and ethanol productivity (2.72 g/L/h) but still 34 % of xylose consumption after detoxification.

**Table 1:** Simultaneous saccharification and ethanol fermentation (SSF) using dry dilute acid pretreated and biodetoxified corn stover feedstock with changing biodetoxification time

Detoxification time (day)	Inhibitors in hydrolysate (g/L)						Sugars in hydrolysate^a^ (g/L)		Ethanol titer (g/L)	Ethanol productivity (g/L/h)	Ethanol yield^b^ (%)
	Furfural	HMF	Acetate	Vanillin	4-HBA	Syringaldehyde	Glucose	Xylose			
0	0.75	0.53	6.16	0.57	0.06	0.25	41.99	44.65	5.31	0.39	5.97
1	0.35	0.44	5.88	0.45	0.03	0.19	71.57	48.65	46.16	1.27	58.34
2	0.16	0.24	4.99	0.22	0.02	0.06	71.52	45.19	46.85	1.60	57.08
3	0.07	0.20	4.07	0.03	0.02	0.03	74.27	44.24	47.88	2.12	57.28
4	0.00	0.00	1.28	0.09	0.02	0.05	81.13	29.58	50.00	2.72	59.63
5	0.00	0.00	1.37	0.08	0.02	0.05	79.28	28.58	48.19	2.90	55.70
7	0.00	0.00	1.35	0.08	0.02	0.06	82.47	23.07	51.07	2.62	57.29
9	0.00	0.00	0.75	0.02	0.02	0.03	81.25	14.61	50.41	2.36	55.30
12	0.00	0.00	1.63	0.02	0.01	0.03	83.01	10.49	52.33	2.66	57.30
15	0.00	0.00	0.80	0.01	0.01	0.02	91.31	12.10	53.88	2.67	59.38

SSF was initiated by pre-hydrolysis for 12 h and then the adapted *S. cerevisiae* DQ1 was inoculated into the hydrolysate at 10 % inoculum to start SSF. SSF conditions: 30 % solids loading, 15 FPU/g DM cellulase dosage, 150 rpm agitation rate; Pre-hydrolysis was conducted at 50 °C, pH 4.8, and SSF was conducted at 37 °C, pH 5.5, respectively

^a^Glucose and xylose concentration were detected in the hydrolysate after 12 h’ enzymatic hydrolysis by cellulase

^b^Ethanol yield was calculated using the methods described by Zhang [41]

### Accelerated detoxification of *A. resinae* ZN1 under aeration and well-mixing conditions

**Fig. 2:**
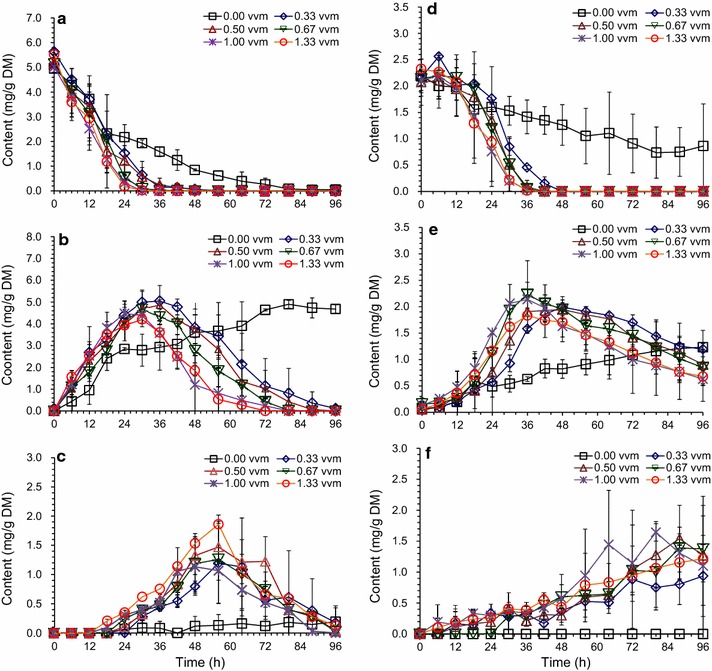
Bioconversions of furfural and HMF on pretreated corn stover in aerated biodetoxification mode. Aeration rate was set to 0.00, 0.33, 0.50, 0.67, 1.00, and 1.33 vvm (defined as the air volumetric flowrate in liter per minute to the corn stover feedstock volume in liter). Biodetoxification conditions: 28 °C, pH 5.5, 10 % inoculum. **a** Furfural; **b** Furfuryl alcohol; **c** Furoic acid; **d** HMF; **e** HMF alcohol; **f** HMF acid

**Fig. 3:**
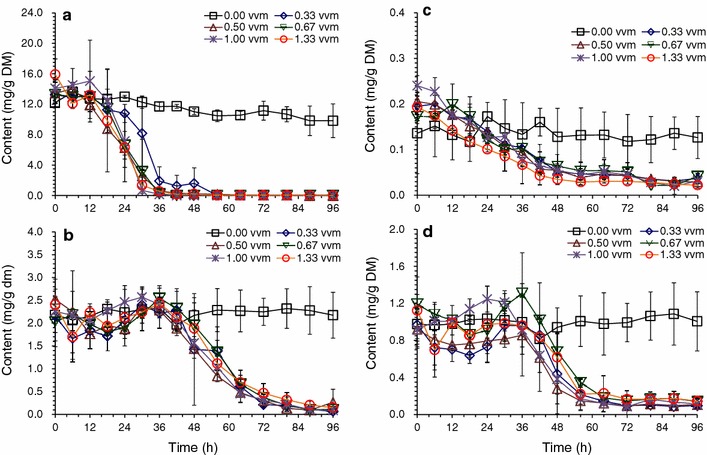
Bioconversions of acetic acid and phenolic compounds on pretreated corn stover in aerated biodetoxification mode. Same biodetoxification conditions with Fig. 2. **a** Acetic acid; **b** Vanillin; **c** 4-Hydroxybenzaldehyde (4-HBA); **d** Syringaldehyde

**Fig. 4:**
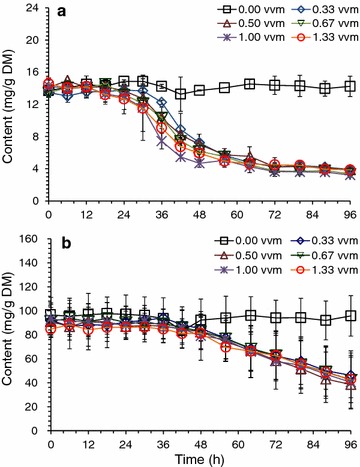
Sugar consumption on pretreated corn stover in aerated biodetoxification mode. Same biodetoxification conditions with Fig. 2. **a** Glucose; **b** Xylose

Aeration was found to accelerate the biodegradation rate of inhibitor compounds in liquid medium in our previous study [28]. In this study, aeration was applied to the conventional solid state biodetoxification of the CS prepared by dry dilute acid pretreatment in well-mixing bioreactor with helical ribbon stirrer agitator. The results in Figs. 2 and 3 show the accelerated degradation of furfural, HMF, and other inhibitors in the biodetoxification under the aeration rate of 0.33 vvm compared to the batch without aeration in the fermenter, which indicated that the aeration was an effective way to improve the efficiency of biodetoxification by *A. resinae* ZN1. The conversion of furfural to furfuryl alcohol then to furoic acid was accelerated by increasing aeration rate in the range of 0.33–1.33 vvm (Fig. 2a–c). The conversion of HMF to HMF alcohol and HMF acid was accelerated only when furfural was reduced to the half of original content (Fig. 2d–f). Similar to HMF conversion, the conversions of acetic acid were accelerated when furfural was reduced to a low content (Fig. 3a). The content of vanillin and syringaldehyde increased a little and then quickly decreased when furfural had been completely removed (Fig. 3b, d), which was similar with the phenomenon described in Fig. 1d. Figure 4 showed that no obvious sugar (mainly xylose and minor glucose) consumption was detected in the batch without aeration, which was opposite to the results of the culture in plastic boxes during static biodetoxification. The sealed structure of fermenter made the detoxification process in an anaerobic environment, which the fungus *A. resinae* ZN1 stop the inhibitor degradation. Sugars did not really start to be consumed by* A. resinae* ZN1 when furfural was not completely converted even under the increasing aeration rate (Fig. 4). Most glucose in the first 24 h and most xylose in the first 48 h still remained unchanged until furfural was completely removed. In another word, glucose and xylose were reserved during the aerated biodetoxification period. The acceleration in the biodetoxification and reservation of sugars in the pretreated CS under aeration were similar to that in the synthetic medium described by Ran et al. [28]. Furthermore, the acceleration in the biodetoxification of pretreated CS was caused by the faster conversion furfural to furfuryl alcohol (Figs. 2 and 3), which indicated that the step-limiting step during biodetoxification under aeration was also the furfural conversion. As an ending indicator of biodetoxification, furfural was completely converted within 36 h at the aeration rate of 1.00 vvm from 96 h of the non-aerated conventional biodetoxification.

Furfural as a volatile compound could be easily taken away under the constant aeration condition, which made it hard to evaluate if the improved detoxification was caused by the biological way of *A. resinae* ZN1. To further confirm the effect of detoxification by *A. resinae* ZN1, the control experiments using pretreated CS without inoculation of *A. resinae* ZN1 was carried out under different aeration as shown in Additional file 1: Figure S1. The volatilization of furfural was similar among the batch under the aeration rate of 0.33, 0.50, 0.67 vvm. The furfural content was reduced to 50 % of the origin content under these three aeration rate after 36 h. The increase of the furfural volatilization could be observed when the aeration rate was increased to 1.00 and 1.33 vvm, where about 70 % of furfural was removed by aeration. However, there is no generation of furfuryl alcohol and furoic acid during the process. So was the degradation of HMF. These results were in accordance with that in the synthetic medium with single inhibitor addition described by Ran et al. [28]. As mentioned above, furfural conversion was the first step for biodetoxification and the corresponding furfuryl alcohol and acid would be generated as the metabolites of furfural by *A. resinae* ZN1 in a corresponding concentration. Thus, the inhibitor degradation in the pretreated CS was due to the biological detoxification by *A. resinae* ZN1.

**Fig. 5:**
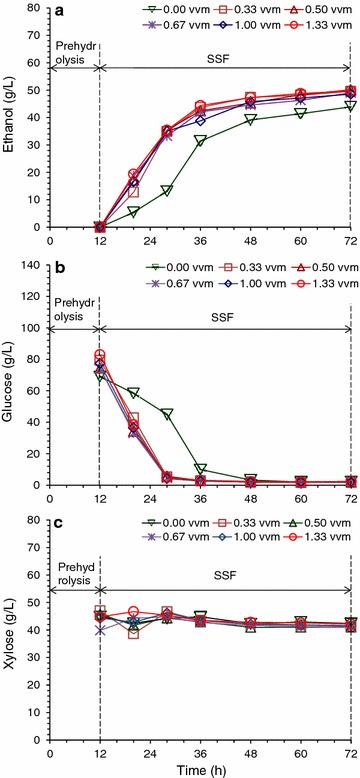
SSF of pretreated corn stover feedstock after accelerative biodetoxification. The dry dilute acid pretreated corn stover was biodetoxified for 36 h at 1.00 vvm of aeration. **a** Ethanol generation; **b** Glucose consumption; **c** Xylose consumption. SSF was initiated by pre-hydrolysis for 12 h and then the adapted *S. cerevisiae* DQ1 was inoculated into the hydrolysate at 10 % inoculum to start SSF. SSF conditions: 30 % solid loading, 15 FPU/g DM cellulase dosage, 150 rpm agitation rate. Pre-hydrolysis was conducted at 50 °C, pH 4.8, and SSF was conducted at 37 °C, pH 5.5

The consequent SSF using the aerated biodetoxified CS feedstock showed obvious advantage in ethanol titer, productivity, and glucose consumption rate over the conventionally detoxified feedstock (Fig. 5). At aeration rate of 1.00 vvm for 36 h, the ethanol titer of 48.56 g/L and ethanol productivity of 2.07 g/L/h were similar to that under the conventional detoxification for 96 h. Increased aeration rate from 0.33 to 1.33 vvm gave approximately the same results, indicating that the minimum aeration rate was sufficient enough for aerated biodetoxification. Xylose concentration was still maintained at 40–50 g/L using the aerated detoxified CS, comparing to only 10–20 g/L of xylose in the conventional detoxification. The saved xylose could be used for producing more ethanol when xylose utilizing strain was used. Xylose maintaining from biodetoxification process made it possible for xylose extraction from ethanol broth. Moreover, the xylose maintaining makes the dry dilute acid pretreatment and biodetoxification applicable for the process of the products fermented by various xylose fermenting strains, which is meaningful for the biorefinery from lignocellulose in the future. Thus the accelerated biodetoxification by *A. resinae* ZN1 was defined as the fast inhibitor degradation process occurring on pretreated lignocellulose feedstock by *A. resinae* ZN1 under proper aeration.

**Fig. 6:**
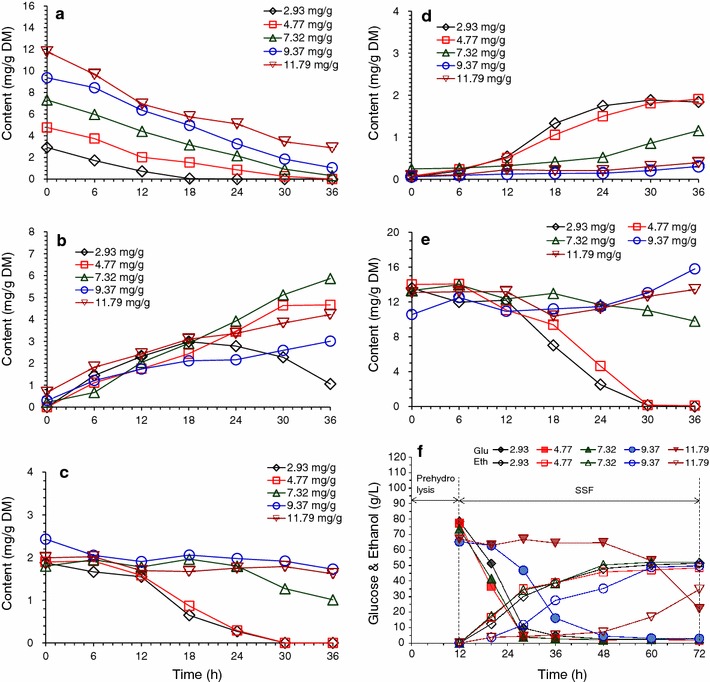
Accelerative biodetoxification of pretreated corn stover feedstock at changing furfural content. **a** Furfural; **b** Furfuryl alcohol; **c** HMF; **d** HMF alcohol; **e** Acetic acid; **f** SSF at 30 % solids loading and 15 FPU/g DM cellulase dosage. Furfural content in the pretreated corn stover was adjusted to 2.93, 4.98, 7.32, 9.37, and 11.79 mg/g DM. Biodetoxification conditions: 28 °C, pH 5.5, 1.00 VVM aeration rate. Agitation was switched on before taking samples

To examine the flexibility of aerated biodetoxification at different pretreatment severity, CS with additionally added furfural was regulated to generate a furfural gradient of 2.93, 7.32, 9.37, and 11.79 mg/g DM, then biodetoxified under aeration condition and sent to SSF. The conversion rate of furfural by *A. resinae* ZN1 was approximately the same at different furfural concentration, but the time used for complete consumption increased with increasing furfural concentration (Fig. 6a). Furfural was unable to be completely degraded when furfural was above 10 mg/g DM in the pretreated CS. Conversion of furfural to furfuryl alcohol and furoic acid was also inhibited by higher furfural content (Fig. 6b). Although HMF and acetic acid content were same to the control feedstock, high furfural also decreased their conversion rates (Fig. 6c–e). The SSF result shows that the ethanol titer, productivity, and glucose consumption rate were significantly affected when furfural content increased to about 10 mg/g DM (Fig. 6f). When furfural was reduced to about 7.0 mg/g DM in the pretreated CS (equivalent to 6.0 g/L in the hydrolysate), the 36 h’ aerated biodetoxification was still able to let SSF at a satisfactory performance. Compared to furfural tolerant stains *Leuconostoc* strains and *Enterobacter cloacae* GGT036 researched by Hunter et al. [13] and Choi et al. [15], *A. resinae* ZN1 possesses the stronger degradation capacity and higher tolerance to furfural, which made it capable to detoxify extensively pretreated lignocellulose feedstock. Besides the furfural adaption, *A. resinae* ZN1 also had a wide adaption on the inhibitors generated from pretreatment process. Compared to the biodetoxification by bacteria and yeast, the fungus had an advantage on the ability to degrade the various kinds of inhibitors generated in the pretreatment process due to the more complete metabolic pathway. The fungus *Coniochaeta lignaria* isolated by Nichols et al. [21] also showed great ability on various inhibitor degradation in the hydrolysate within 24 h with the aid of high rotation rate in the shaker. Compared to the research and application of *Coniochaeta lignaria* [20–23], *A. resinae* ZN1 could handle a much higher content of inhibitors in the solid CS with 50 % water content, which removed the water wash and the separation of hydrolysate after pretreatment step. These properties made *A. resinae* ZN1 and accelerated biodetoxification step potential in the biorefinery process at a large scale or industrial production.

### Cell growth of *A. resinae* ZN1 during accelerated biodetoxification on pretreated corn stover

**Fig. 7:**
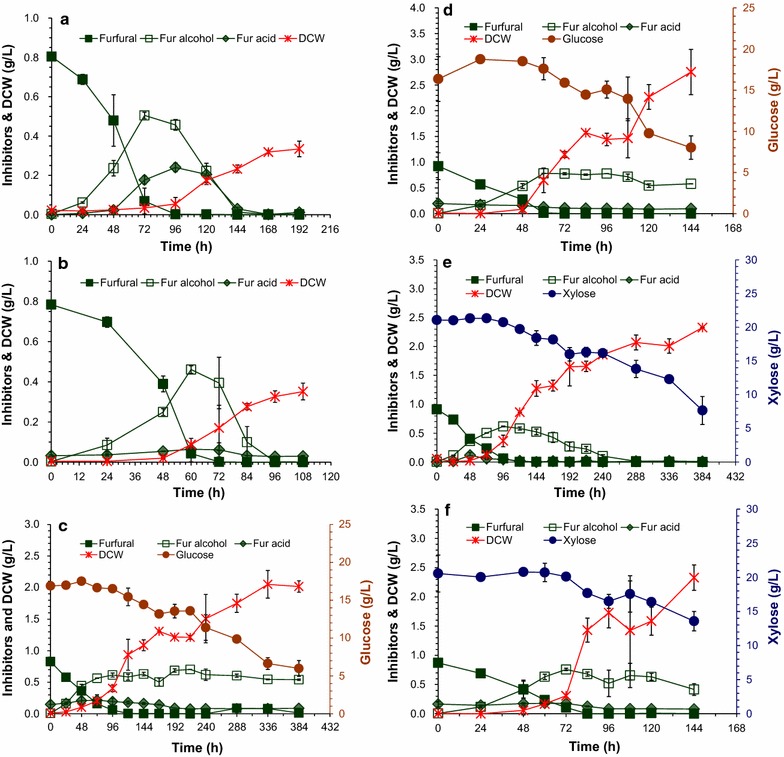
Cell growth of *A. resinae* ZN1 in liquid synthetic medium. 1 g/L of furfural was added into the medium. The culture was carried out at 28 °C in static incubator and shaker. **a** In the static incubator with furfural as a sole carbon source; **b** In the shaker under 100 rpm with furfural as a sole carbon source; **c** In the static incubator with 20 g/L glucose as carbon source; **d** In the shaker under 100 rpm with 20 g/L glucose as carbon source; **e** In the static incubator with 20 g/L xylose as carbon source; **f** In the shaker under 100 rpm with 20 g/L xylose as carbon source

Oxygen supply by aeration also enhances the cell growth of *A. resinae* ZN1 thus increases cell mass as biocatalyst for improvement of inhibitor conversion. In this study, we firstly measured the cell growth of *A. resinae* ZN1 in the liquid medium by monitoring the change of dry cell weight (DCW) (Fig. 7). At static culture condition, the cells grew to about 0.3 g/L when 0.8 g/L of furfural was used as sole carbon (Fig. 7a), and to about 2 g/L in the medium when 20 g/L of glucose (Fig. 7c) or xylose (Fig. 7e) were added to the medium. When the cell culture was moved to the shakers to increase dissolved oxygen in the medium, the conversion period of furfural was reduced by about 36 h compared to that in the culture in static state (Fig. 7b, d, f). However, the cell growth was still slow during first 60 h (furfural degradation) and then increased quickly to the similar DCW value (2 g/L) to that in the static culture within a much shorter time (from 384 to 144 h). These results indicate that the increased dissolved oxygen did not obviously improve the cell growth of *A. resinae* ZN1, but accelerated the furfural conversion.

**Fig. 8:**
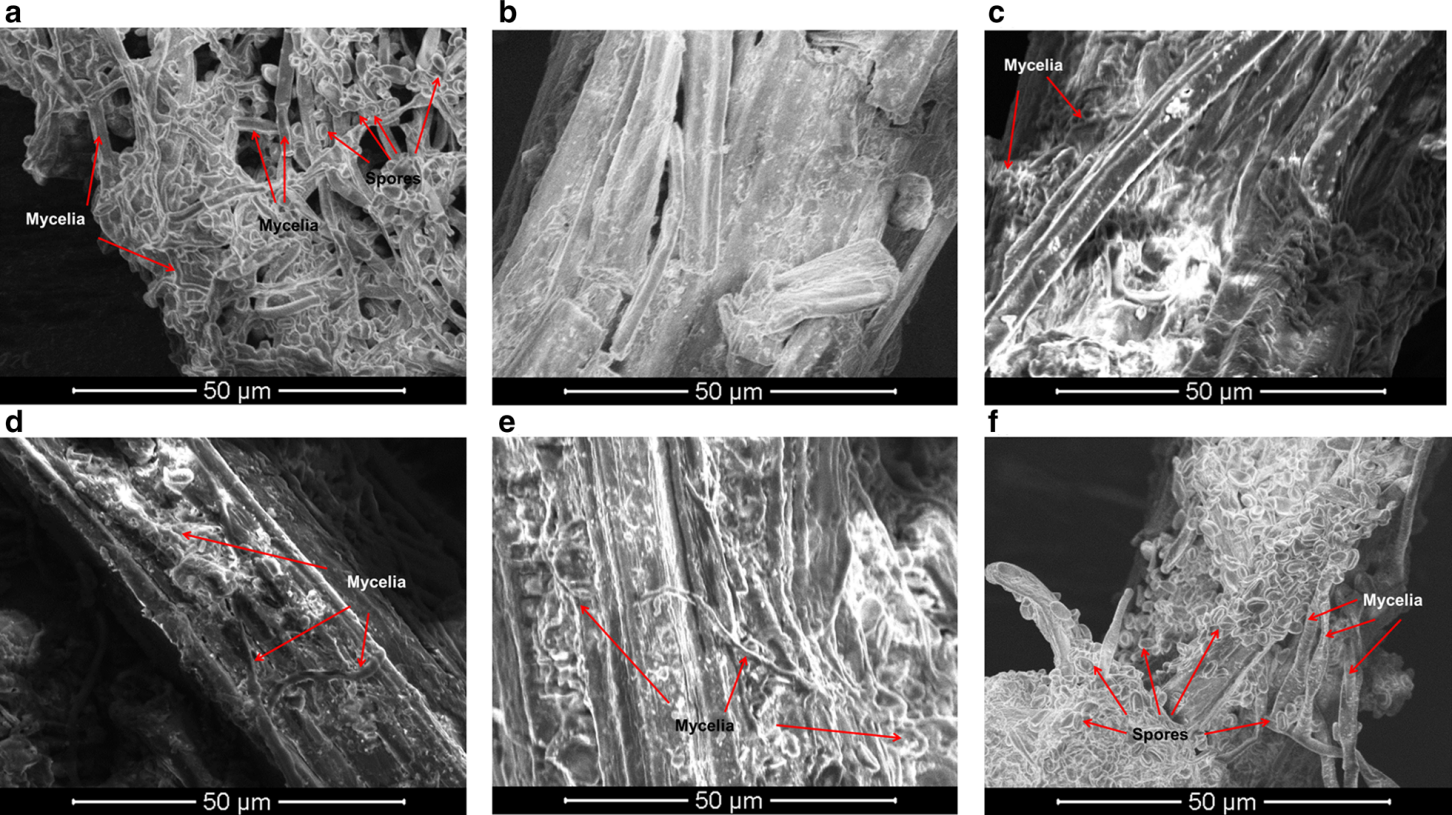
Cell growth of *A. resinae* ZN1 on pretreated corn stover during aerated biodetoxification by Environmental Scanning Electric Microscope (ESEM). **a** *A. resinae* ZN1 growing on PDA slant; **b** Corn stover without inoculum of *A. resinae* ZN1; **c** Corn stover inoculated by *A. resinae* ZN1 at 0 h; **d** Corn stover inoculated for 18 h; **e** Corn stover inoculated for 36 h; **f** Corn stover inoculated for 96 h

When the measurement of cell growth on the solid pretreated CS was tested, we found that the routine quantitative methods did not work. Obviously the direct DCW measurement was not suitable because cell mycelia were firmly mixed with solid lignocellulose fibers. On the other hand, the routine glucosamine detection method for fungus mycelia assay [30] and the conservative *β*-actin expression detection by real-time fluorescence quantitative polymerase chain reaction [31] were tested, but also did not work. The reason is that pretreatment disrupted the CS cell wall then the glucosamine or *β*-actin proteins from CS cells were exposed, and were not able to distinguish from the similar proteins in *A. resinae* ZN1. As a qualitative alternative method, the direct observation of fungus mycelia and spores using environmental scanning electron microscope (ESEM) was applied (Fig. 8). *A. resinae* ZN1 fungus on potato dextrose agar slant (PDA slant) was used as the positive control (Fig. 8a) and the CS solids without *A. resinae* inoculation were used as the negative control (Fig. 8b). After the inoculation of *A. resinae* cells, cell mycelia were observed on CS solids (Fig. 8c). The mycelium numbers were not in an observable increase after aerated detoxification for 18 h (Fig. 8d). Limited increase of mycelia was observed when aerated biodetoxification lasted for 36 h (Fig. 8e). Only when the biodetoxification extended to 96 h, the massive growth of mycelia was observed, which was similar to the seed of *A. resinae* ZN1 on the PDA slant (Fig. 8f). The results suggest that similar to the liquid medium, the cell growth of *A. resinae* ZN1 during inhibitors’ degradation was limited even though sufficient oxygen was supplied.

**Fig. 9:**
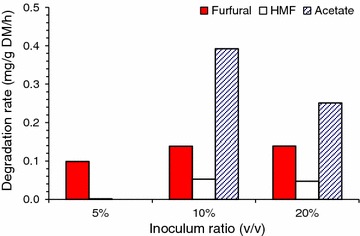
Accelerative biodetoxification of pretreated corn stover with increasing inoculum ratio of *A. resinae* ZN1. The data were detected at 36 h’ detoxification. Detoxification conditions: 28 °C, pH 5.5, 1.00 vvm aeration rate, agitation was switched on before taking samples

To further evaluate the fungus growth during aerated biodetoxification, biodetoxification was conducted with changing inoculum under aeration condition (Fig. 9). The conversion rate of inhibitors was reduced when the inoculum ratio decreased from 10 to 5 % indicating that the amount of initial strain inoculated into the pretreated CS had a significant impact on the biodetoxification. Also the results showed that the growth during the inhibitor degradation was slow because no more strains could help to improve the efficiency of inhibitor degradation at a low initial strain. These results go well with the phenomenon observed by ESEM (Fig. 8). There is no further increase when inoculum ratio of *A. resinae* spores increased from 10 to 20 % (Fig. 9). Perhaps 10 % inoculum ratio was the minimum for cell consortium growth with the current pretreatment severity but the further increase of spores was not helpful to accelerating the biodetoxification rate.

Since the cell growth of *A. resinae* strain was not improved by aeration, the accelerated biodetoxification rate should come from the enhanced inhibitor metabolism of the same amount of cell mass. From the metabolic analysis of furfural and HMF in *A. resinae* (Ran et al., 2014), the reductive cofactors such as nicotinamide adenine dinucleotide or nicotinamide adenine dinucleotide phosphate were generated when furfuryl alcohol was converted to furfural and then to furoic acid. The increased cofactor supply could further support the bioconversion of HMF [11, 16]. Acetic acid could be directly converted to acetyl-CoA with the aid of ATP [32] and then enter the TCA cycle. The fast degradation of acetic acid and no metabolites detected suggested that the metabolism of acetic acid in *A. resinae* ZN1 was similar to that described by Wei et al. [32]. The accelerated conversion of furfural, furfuryl alcohol, and furoic acid provide the NADH or NADPH which could be converted to ATP for the fast degradation of acetic acid. The metabolic pathway of the three main phenolic compounds was similar to furfural and HMF, where the phenolic aldehydes were converted to their alcohol form and acid form. The formation of furan acids could provide reducing power for phenolic compound degradation [33]. In this study, the accelerated conversion of furfuryl alcohol to furoic acid by aeration could have played an initiating power for the overall acceleration of biodetoxification of all inhibitor compounds. However, to confirm the hypothesis, the quantitative assay of NADH or NADPH in *A. resinae* cells is needed under the condition of clear separation of fungus mycelia from lignocellulose fibers in the future studies.

## Conclusions

The accelerated biodetoxification realized by aeration and well mixing greatly shortened the period of inhibitor removal from pretreated CS by *A. resinae* ZN1 and ensured the sequent ethanol production. The retention of xylose component generated during pretreatment made the process more efficient and economic. The improvement of the conversion NAD and NADH involved in the metabolism of furan compounds under aeration condition enhances the biodetoxification rather than the growth of *A. resinae* ZN1. The accelerated biodetoxification provides a practical solution to increase the efficiency of ethanol production from lignocellulosic materials.

## Methods

### Raw materials and pretreatment

Corn stover (CS) was harvested in fall 2012 from Dancheng, Henan, China. Corn stover was washed to remove field dirts, sand, and metals then dried and sealed in plastic bags until use. The virgin CS contained 36.18 % of cellulose and 19.83 % of xylan determined by the method of two-step acid hydrolysis described as national renewable energy laboratory protocols [34, 35].

The dry dilute acid pretreatment at about 70 % solid loading was carried out in a helical ribbon impeller-driven reactor by co-currently feeding of dilute sulfuric acid solution and CS as described by Zhang et al. [36] and He et al. [37]. The pretreatment condition was fixed at 175 °C for 5 min at 2.5 % sulfuric acid usage per dry CS. The pretreated CS obtained from the reactor maintained the solid state with about 45 % solid content. The pretreated CS contained 40.14 % of cellulose, 3.08 % of xylan, 13.45 mg/g DM of glucose, 97.78 mg/g DM of xylose, 13.28 mg/g DM of acetic acid, 5.34 mg/g DM of furfural, 2.14 mg/g DM of HMF, 2.26 mg/g DM of vanillin, 0.24 mg/g DM of 4-HBA, 0.92 mg/g DM of syringaldehyde determined by the method of two-step acid hydrolysis as described in NREL protocols [34, 35].

### Enzyme and reagents

The cellulase enzyme Youtell No.6 was purchased from Hunan Youtell Biochemical Co., Yueyang, Hunan, China. The filter paper and cellobiase activity of the enzyme were 135 FPU/g and 344 CBU/g measured by the methods described by NREL LAP-006 [38] and Ghose [39], respectively. The protein content in the cellulase was 90 mg/g measured by Bradford method. Chemical reagents used in the experiments were purchased from Shanghai Lingfeng Reagents Co. and Shanghai Demo Medical Tech Co., Shanghai, China.

### Strains and culture

The biodetoxification strain *A. resinae* ZN1 (CGMCC 7452, Chinese General Microorganisms Collection Center, Beijing, China) was used for removal of inhibitors from pretreated CS materials. Spore suspension of *A. resinae* ZN1 was inoculated into the liquid medium containing 1 g/L of furfural, 2 g/L of KH_2_PO_4_, 1 g/L of (NH_4_)_2_SO_4_, 1 g/L of MgSO_4_·7H_2_O, 1 g/L of yeast extracts, 0.5 g/L of CaCl_2_, and 20 g/L of glucose or xylose, then cultured in the incubator or shaker at 28 °C, and constant pH value of 5.0 for 16 days.

The ethanol fermenting strain *Saccharomyces cerevisiae* DQ1 (CGMCC 2528, Chinese General Microorganisms Collection Center, Beijing, China) was used for ethanol fermentation. *S. cerevisiae* DQ1 was evolutionarily adapted for 65 days in freshly pretreated CS hydrolysate and stored at −80 °C till use [25]. Then the yeast was first cultured in the synthetic medium containing 20 g/L of glucose, 2 g/L of KH_2_PO_4_, 1 g/L of (NH_4_)_2_SO_4_, 1 g/L of MgSO_4_·7H_2_O, 1 g/L of yeast extracts, and then cultured in the hydrolysate of CS as seed culture according to the procedure as described in Chu et al. [40].

### Solid state biodetoxification

The pretreated CS was first neutralized by 20 % (*w/w*) Ca(OH)_2_ slurry to pH 5.5 and then inoculated by *A. resinae* ZN1 in the form of mycelia on CS solids at 10 % inoculum by weight. The process was carried out in a sealed plastic box without aeration. The mixture of inoculum and pretreated CS occupied 1/4 volume of the box. No additional nutrients were supplied on the pretreated CS material. The detoxified CS was collected as feedstock for consequent SSF. Five grams of sample was withdrawn periodically from the culture and mixed with 50 g water under 30 °C, 180 rpm for 1 h. The supernatant of the mixture was collected for inhibitors and sugars’ analysis.

Fast biodetoxification of pretreated CS was conducted in 5 L bioreactor equipped with helical ribbon impeller as described by Zhang et al. [42]. 900 g pretreated CS was loaded in the reactor with 10 % (*w/w*) inoculum of seed culture. Biodetoxification was then carried out under different aeration rate of 0, 0.33, 0.50, 0.67, 1.00, and 1.33 vvm. The aeration rate (vvm) was defined as the air flow rate (L/min) per liter of loosely packed pretreated CS material in the bioreactor. The CS material was mixed slowly for 90 s prior to the samples withdrawing and the rest time of biodetoxification was still carried out in a solid state culture.

### Simultaneous saccharification and ethanol fermentation (SSF)

Simultaneous saccharification and ethanol fermentation using the detoxified CS as feedstock was carried out at 30 % (*w/w*) solid loading, 15 FPU/g DM cellulase dosage, 150 rpm, and pH 5.5 in the 5 L helical ribbon bioreactor [41]. 10 % inoculum of the yeast seed culture and the nutrients (2 g/L of KH_2_PO_4_, 1 g/L of (NH_4_)_2_SO_4_, 1 g/L of MgSO_4_·7H_2_O, 1 g/L of yeast extract) were added into the slurry after 12 h’ pre-hydrolysis. Temperature was controlled at 50 °C during 12 h’ pre-hydrolysis and 37 °C during SSF. When the rapid detoxification was carried out in the 5 L helical ribbon bioreactor, the following SSF was then initiated in the same reactor. The ethanol yield was calculated using the modified equation for the ethanol fermentation at high solids loading described in [42]. The equation was described as below: $\text{Ethanol yield}\,\, ({\%})= \frac{{[C]}\times{W}} {976.9 - 0.804\times{[C]}}\times\frac{1}{0.511\times{f}\times{\text{[biomass]}}\times{m}\times{1.111}}\times{100}\, {\%}$ where [*C*] is the concentration of ethanol in the fermentation broth at the end of the SSF (g/L), *W* is total water input into the SSF system (g), *f* is the cellulose content in the dry corn stover (g/g), [biomass] is the dry corn stover loading (g/g), m is total weight of the SSF system at the beginning of the operation, 976.9 is the correction factor, 1.111 is theconversion factor for cellulose to equivalent glucose, 0.511 is the conversion factor for glucose to ethanol based on the stoichiometric biochemistry of yeast.

### Fungus growth assay

Cell growth of *A. resinae* ZN1 in liquid medium was measured by DCW. The culture broth was first filtered and the cell mycelia were dried at 105 °C for 12 h till constant weight.

Cell growth of *A. resinae* ZN1 on pretreated CS was qualitatively monitored by ESEM (Quanta 250, FEI Co., USA) with accelerated voltage of 12.5 kV. Fungal filamentous mycelia and spores observed from ESEM were used for illustration of fungus growth.

### Inhibitors, sugars, and ethanol analysis

Sugars, organic acids, and ethanol were analyzed on HPLC (LC-20AD, Shimazu, Kyoto, Japan) equipped with a Bio-rad Aminex HPX-87H column (Bio-rad, USA) and RID-10A detector (Shimadzu, Kyoto, Japan). 5 mM H_2_SO_4_ solution was used as flow phase at the flow rate of 0.6 ml/min. Furans were analyzed on HPLC (LC-20AT, Shimazu, Kyoto, Japan) equipped with a YMC-Pack ODS-A column (YMC, Tokyo, Japan) and an SPD-20A UV detector (Shimadzu, Kyoto, Japan).

Furfural and furfuryl alcohol were analyzed using 50 % acetonitrile as flow phase at the flow rate of 1.0 ml/min at the column temperature of 35 °C and the detection wavelength of 220 nm. HMF and HMF alcohol were analyzed using the following gradient: the initial flow phase was composed by pure water (pump A) and acetonitrile (pump B) at a ratio of 95 to 5 %; first, acetonitrile was increased from 5 to 100 % over 0–15 min; then, acetonitrile was decreased from 100 to 5 % over 15–20 min; finally, acetonitrile was used at 5 % over 20–30 min. Phenolic compounds were analyzed using the following gradient: the initial flow phase was composed by 0.1 % formic acid solution (pump A) and acetonitrile (pump B) at a ratio of 90 to 10 %; first the acetonitrile concentration increased to 35 % over 4–5 min and kept at 35 % over 5–20 min; then the acetonitrile concentration decreased to 10 % over 20–21 min; finally, acetonitrile was used at 10 % over 21–30 min. All samples were filtered through 0.22 μm membrane prior to HPLC analysis.

## Additional file

10.1186/s13068-016-0438-9 The detection of furfural volatilization without inoculum under different aeration rate in the 5 L fermenter. The volatilization of furfural in the pretreated corn stover under different aeration rate without inoculation of *A. resinae* ZN1. The conditions were controlled at 28 °C, pH 5.5. The volatilization was calculated by the ratio of reduced furfural content to the original furfural content.

## Abbreviations

HMF: 5-Hydroxymethylfurfural
HMF alcohol: 2,5-Furandiyldimethanol (5-Hydroxymethylfurfuryl alcohol)
HMF acid: 5-Hydroxymethyl-2-furoic acid (5-Hydroxymethylfuroic acid)
NADH: nicotinamide adenine dinucleotide (reduced)
NADPH: nicotinamide adenine dinucleotide phosphate (reduced)
PCR: polymerase chain reaction
ESEM: environmental scanning electron microscope
PDA: potato dextrose agar
NREL: national renewable energy laboratory
LAP: laboratory analytical procedure
