# Supplementary material for: Acceleration of biodetoxification on dilute acid pretreated lignocellulose feedstock by aeration and the consequent ethanol fermentation evaluation
Source: Biotechnol Biofuels. 2016 Jan 26;9:19. doi: 10.1186/s13068-016-0438-9 (PMC4727304; doi:10.1186/s13068-016-0438-9)
Supplement: Supplementary file 1 — 10.1186/s13068-016-0438-9 The detection of furfural volatilization without inoculum under different aeration rate in the 5 L fermenter. The volatilization of furfural in the pretreated corn stover under different aeration rate without inoculation of A. resinae ZN1. The conditions were controlled at 28 °C, pH 5.5. The volatilization was calculated by the ratio of reduced furfural content to the original furfural content. [file 13068_2016_438_MOESM1_ESM.docx]

Figure S1. The volatilization of furfural in the pretreated corn stover under different aeration rate without inoculation of *A. resinae* ZN1. The conditions were controlled at 28 ^o^C, pH 5.5. The volatilization was calculated by the ratio of reduced furfural content to the original furfural content.
